# An Ensemble Learning Model for COVID-19 Detection from Blood Test Samples

**DOI:** 10.3390/s22062224

**Published:** 2022-03-13

**Authors:** Olusola O. Abayomi-Alli, Robertas Damaševičius, Rytis Maskeliūnas, Sanjay Misra

**Affiliations:** 1 Department of Software Engineering, Kaunas University of Technology, 51368 Kaunas, Lithuania; olusola.abayomi-alli@ktu.edu; 2 Department of Multimedia Engineering, Kaunas University of Technology, 51368 Kaunas, Lithuania; rytis.maskeliunas@ktu.lt; 3 Department of Computer Science and Communication, Ostfold University College, 3001 Halden, Norway; ssopam@gmail.com

**Keywords:** diagnostic model, blood tests, COVID-19, deep learning, ensemble learning, small data

## Abstract

Current research endeavors in the application of artificial intelligence (AI) methods in the diagnosis of the COVID-19 disease has proven indispensable with very promising results. Despite these promising results, there are still limitations in real-time detection of COVID-19 using reverse transcription polymerase chain reaction (RT-PCR) test data, such as limited datasets, imbalance classes, a high misclassification rate of models, and the need for specialized research in identifying the best features and thus improving prediction rates. This study aims to investigate and apply the ensemble learning approach to develop prediction models for effective detection of COVID-19 using routine laboratory blood test results. Hence, an ensemble machine learning-based COVID-19 detection system is presented, aiming to aid clinicians to diagnose this virus effectively. The experiment was conducted using custom convolutional neural network (CNN) models as a first-stage classifier and 15 supervised machine learning algorithms as a second-stage classifier: K-Nearest Neighbors, Support Vector Machine (Linear and RBF), Naive Bayes, Decision Tree, Random Forest, MultiLayer Perceptron, AdaBoost, ExtraTrees, Logistic Regression, Linear and Quadratic Discriminant Analysis (LDA/QDA), Passive, Ridge, and Stochastic Gradient Descent Classifier. Our findings show that an ensemble learning model based on DNN and ExtraTrees achieved a mean accuracy of 99.28% and area under curve (AUC) of 99.4%, while AdaBoost gave a mean accuracy of 99.28% and AUC of 98.8% on the San Raffaele Hospital dataset, respectively. The comparison of the proposed COVID-19 detection approach with other state-of-the-art approaches using the same dataset shows that the proposed method outperforms several other COVID-19 diagnostics methods.

## 1. Introduction

The Internet of Things (IoT) and smart home technologies enable the monitoring of people in their homes without interfering with their daily routines [1]. Advances in artificial intelligence (AI) and machine learning (ML) can enable faster patient monitoring, management, and treatment, as well as convert a hospital-only treatment pathway into cost-effective combined home-hospital or even outpatient alternatives, improving overall quality of health care and paving the way for personalized medicine [2]. Digital health signals recorded at home by sensors provide a wealth of clinical data. Such data could be sent to cloud computing infrastructure and analyzed remotely, which is especially useful in the case of various contagious diseases, such as the coronavirus one [3]. However, analyzing real-time data collected from heterogeneous IoT sensors presents several challenges because the data contain significant artifacts because of transmission and recording limitations, are highly imbalanced and incomplete because of subject variability and resource limitations, and involve multiple modalities [4].

Currently, the COVID-19 SARS-CoV-2 coronavirus pandemic has hit the world with more than 400 million confirmed cases and nearly 6 million deaths recorded. It continues to have numerous negative consequences on health, society, and the environment [5]. The gold standard measure is the amplification of viral RNA by reverse transcription polymerase chain reaction (rRT-PCR) [6]. However, it presents established weaknesses: lengthy processing times (3–4 h to deliver results), possible reagent shortages, insufficient RT-PCR test kits, high demand for experts [7], false negative rates of 15–20%, and the requirement for accredited laboratories, costly infrastructure, and qualified workers. Recently, the CDC (Centers for Disease Control and Prevention) withdrew the Emergency Use Authorization (EUA) of the 2019-Novel Coronavirus (2019-nCoV) Real-Time RT-PCR Diagnostic because of its inability to differentiate between the SARS-CoV-2 and influenza viruses. Therefore, reliable new substitute tests, quicker, less costly, and more open tests, are required [8].

More attention has been paid to investigating the potential of state-of-the-art AI and ML methods to tackle COVID-19 (see the reviews of the methods in [9,10]). The focus of research endeavors is to aid in the diagnosis and prediction of diseases, detection, care, control, monitoring of diseases, the development of antiviral drugs [11], and image segmentation techniques [12], as well as to forecast the number of active cases [13] and death cases [14]. We discuss the applications of ML techniques to assist health professionals in the detailed and effective timely identification of COVID-19. A prior indicator of the presence of this virus is provided by the initial screening process, whereas further diagnosis confirms the existence or nonexistence of the virus. The application of ML algorithms using some medical images has provided promising results with examples of images such as computed tomography (CT) scans and X-ray images, while complementing traditional COVID-19 diagnostic strategies using ensemble methods [15], genetic algorithms combined with traditional ML algorithms [16], nature-inspired optimization methods [17], and deep learning methods [18]. However, considering the level of radiation exposure from CT/X-ray scan equipment, the corresponding minimal number of accessible devices and the high cost of these devices make them difficult to use for real-time screening.

Recently, several researchers have suggested the use of ultrasound screening for children and pregnant women to be noninvasive and X-ray radiation-free for the identification of COVID-19 [19]. Other researchers investigated the processing and analysis of voice (speech) [20] and cough [21] for the identification of COVID-19. COVID-19 detection from urine, stool, and feces samples was also considered in several studies [22,23]. Among other biomarkers notable for COVID-19 identification, lymphocytes, cardiac troponin, platelet count, and renal biomarkers have been discussed [24]. In [25], the use of blood tests (hemoglobin, white cells, neutrophil count, and lymphocyte count, platelets, bilirubin, etc.), blood gas results (such as oxygen saturation, and partial pressure of carbon dioxide) and vital signs (such as heart rate, oxygen saturation, and oxygen flow rate) for COVID-19 diagnostics were considered. An early warning system based on scoring vital signs and other variables for predicting the deterioration of the health states of COVID-19 patients was presented in [26]. A variety of clinical trials have recently revealed that the routine blood test parameters of COVID-19 patients indicate substantial differences, and that the detection of these biomarkers can play a crucial role in the initial screening of COVID-19, such as using decision trees [27,28], Random Forest (RF), Naive Bayes (NB), logistic regression (LR), support vector machine (SVM) and k-nearest neighbors (KNN) [28], and SVM [29]. As stated in [30], all the details found in routine blood tests are too strenuous to extract for advanced clinicians.

Recent studies have shown the impact of a critical branch of AI methods, especially the integration of ML algorithms for effective prediction models [31]. However, ML algorithms can learn and discriminate between numerous patterns in the parameters of a routine blood test. In the development of ML algorithms for the identification of COVID-19 from regular blood samples, some initial efforts have begun, as discussed in depth in the next section. This area of research is in the initial research period and requires more attention. Therefore, this study aims to study and compare the performance of different state-of-the-art ML models on a blood sample data set. We applied and evaluated different ML classification algorithms on a different hidden layer. They evaluated the performance of all classifiers using diverse performance metrics.

The major contributions of this paper are highlighted as follows:

1. The proposed algorithm was able to effectively provide the preliminary classification of COVID-19 using relevant feature parameters.
2. The proposed algorithm has a lower computational intensity, and the detection time was in a few seconds.
3. Based on the effectiveness of our proposed model, it can improve pathologist efficiency and aid effective laboratory examination in pathology departments.

The remaining parts of the paper are prepared and sectioned as follows: an extensive review of the literature is discussed in Section 2, while Section 3 presents the framework and detailed description of our proposed methods. The experimental results and the discussion are presented in Section 4, and the last part of the paper is the conclusion and future recommendation, as presented in Section 5. 

## 2. Materials and Methods

This section describes in detail the progress and contributions, including the state-of-the-art methods presented in the previous study on the detection of COVID-19. To further understand the level of the existing study with the contribution of AI methods, especially the ML algorithms used, we reviewed the various literature using blood test results in the detection of COVID-19 with highlights on the methods, contribution, and limitations. 

The authors of [32] evaluated the results of the blood tests to perform an initial screening of likely patients with COVID-19 using the dataset of 598 blood samples from Albert Einstein Hospital, Brazil. The dataset consists of 81 cases of COVID-19. The authors based their experiment on 14 blood features using ML models based on random forest, logistic regression, artificial neural network (ANN), and Lasso elastic-net regularized generalized linear network (GLMNET). The best-performance model gave an accuracy of 87% for ANN. 

A study [33] presented a COVID-19 detection approach based on some ML models, which are XGBoost, LDA, LR, RF, and Decision Tree. The authors investigated the impact of feature/variable selection and dimensionality reduction in features from 12 variables to 4. They concluded that the best accuracies of 89.6% and 85.9% were achieved by XGBoost for 12-variable and 4-variable models, respectively. The later study [25] was conducted using blood test results from Oxford University Hospitals, UK. The XGBoost classifier achieved the accuracy, sensitivity, and specificity of 92.3%, 77.4%, and 95.7%, respectively.

Recent work by [34] carried out an analysis using two ML algorithms in the detection of COVID-19 on routine blood tests. The ML algorithms used by the authors are RF and SVM on a small data set of 294 blood samples obtained from Wuhan Union Hospital and Kunshan People’s Hospital, China. Fifteen characteristics were selected for analysis and the experimental results showed that SVM outperformed random forest classifiers with accuracy, precision, sensitivity, and specificity of 84%, 92%, 88%, and 80%, respectively. 

Five ML algorithms, namely gradient boost trees, neural networks, logistic regression, random forest, and SVM, were proposed by authors in [35] in the diagnosis of COVID-19. A dataset containing a total number of 235 blood samples with 102 established cases of COVID-19 was gathered from Albert Einstein Hospital in Brazil and 15 relevant characteristics were selected. SVM gave the best classification result with very little significance compared to previous work reviewed in this study on AUC, sensitivity, and specificity of 85%, 68%, and 85%, respectively. 

Another dataset consisting of 279 cases from San Raffaele Hospital, Milan, Italy, was analyzed for the early detection of COVID-19 by the authors of Brinati et al. [27]. In the performance of seven ML models such as KNN, DT, NB, extremely randomized trees (ET), LR, RF, and SVM, the experimental results showed that the RF model outperformed other classifiers with an accuracy of 86% and a sensitivity of 95%. 

Feng et al. [36] explored decision trees (DT), LR with Ridge regularization, LR with LASSO, and AdaBoost algorithms for real-time detection of COVID-19 from a set of demographic, clinical signs, biomarkers, vital signs, and blood test values. The dataset contains blood test results gathered from 132 patients (26 positives) from First Medical Center (FMC), Beijing, China. LASSO was used to select 18 features from the original 46 features. The best-performing model was based on LR with LASSO with an AUC, specificity, and sensitivity of 93.8%, 77.8%, and 100%, respectively. 

Another study [37] presented an LR-based ML classifier to detect COVID-19 using three major component counts. The training set consists of 390 cases including established COVID-19 cases from Stanford Health Care and a different dataset was used for validation. 

Further studies from [38] analyzed and applied six state-of-the-art methods including MLP, SVM, RT, NB, RF, and Bayesian Networks (BN). The study was carried out using a dataset consisting of 564 samples, including 559 established COVID-19 samples from Albert Einstein Hospital in Brazil. The authors performed oversampling using the SMOTE technique because of the limited data size and, for feature selection, a manual method and two algorithms based on PSO and evolutionary search were utilized. The performance model with the highest results was obtained from the BN model with an accuracy, precision, specificity, and sensitivity of 95.159%, 93.8%, 93.6%, and 96.8%, respectively.

The authors of [39] presented a neural network model for the detection of the severity of COVID-19 in small data samples from the Tongji Medical College of Huazhong University of Science and Technology, Hubei, in collaboration with the Tumor Center of Union Hospital, China. The authors evaluated the severity of COVID-19 on 151 images after selecting features. 

An extreme gradient boosting (XGBoost) model was applied by the authors in Kukar et al. [40] to identify COVID-19. A total of 5333 blood samples, including 160 established COVID-19 samples, were obtained from the University Medical Center Ljubljana, Slovenia. Thirty-five relevant characteristics were selected for further analysis and the experimental results showed an improved AUC of 97%, 81.9% sensitivity, and a specificity of 97.9%. 

A robust model for oversampling and ensemble learning based on the integration of the SVM and SMOTEBoost methods was proposed in [41]. The results of 10 SVM-SMOTEBoost models were used for the ensemble learning, and the overall performance was determined using the average results of the 10 models. The proposed model was able to achieve an AUC of 86.78%, a sensitivity of 70.25%, and a specificity of 85.98%. 

Aljame et al. [42] proposed an ensemble learning model for the initial screening of patients with COVID-19 from routine blood tests. The model used the dataset obtained from 564 patients of the Albert Einstein Israelita Hospital located in Sao Paulo, Brazil, and achieved an accuracy of 99.88% in discriminating COVID-19 positive cases.

In Wu et al. [43], to identify COVID-19 from a complete blood count, a mixed dynamic ensemble selection (DES) approach for unbalanced data is suggested. This approach combines data preparation with enhanced DES. First, the authors balance the data and reduce noise using the hybrid synthetic minority oversampling approach and edited nearest neighbor (SMOTE-ENN). Second, a hybrid multiple clustering and bagging classifier generation (HMCBCG) approach is presented to enhance the variety and local regional competency of candidate classifiers to improve DES performance. With 99.81% accuracy, HMCBCG with k-nearests oracles eliminate (KNE) achieves the best performance for COVID-19 screening.

AlJame et al. [44] propose a ML prediction model for the diagnosis of COVID-19 based on clinical and regular laboratory data. The model uses an ensemble-based strategy known as deep forest (DF), which employs numerous classifiers in several layers to foster variety and increase performance. The cascade level uses layer-by-layer processing and is made up of three separate classifiers: additional trees, XGBoost, and LightGBM. The DF model has an accuracy of 99.5% on two publicly accessible datasets.

In Babaei Rikan et al. [45], to diagnose positive instances of COVID-19 from three regular laboratory blood test datasets, seven ML, and four deep learning models were presented. To illustrate the relevance among samples, Pearson, Spearman, and Kendall correlation coefficients were used. The suggested models were trained, validated, and tested using a four-fold cross-validation procedure. The deep neural network (DNN) model earned the highest accuracy values in all three datasets.

Buturovic et al. [46] sought to build a blood-based host gene expression classifier for the severity of viral infections, including COVID-19. They created a logistic regression-based classifier for viral infection severity and validated it in a variety of viral infection situations, including COVID-19. In patients with confirmed COVID-19, the classifier exhibited area under curve (AUC) values of 0.89 and 0.87 to detect patients with severe respiratory failure or 30-day mortality, respectively.

In Du et al. [47], several binary classification techniques and classifiers were examined to develop the ML model for illness classification: categorical gradient boosting (CatBoost), support vector machine (SVM), and logistic regression (LR). In three validation datasets, the ML model achieved excellent AUC (89.9–95.8%) and specificity (91.5–98.3%), but low sensitivity (55.5–77.8%) to predict SARS-CoV-2 infection.

Hu et al. [48] proposed a framework based on enhanced binary Harris hawk optimization (HHO) in conjunction with an extreme kernel learning machine (KELM). They used specular reflection learning to improve the original HHO algorithm. The experimental findings reveal that the selected indicators, such as age, partial oxygen pressure, oxygen saturation, sodium ion concentration, and lactic acid, are critical for the early correct evaluation of COVID-19 by the proposed feature selection method.

Kukar et al. [40] built an ML model for the detection of COVID-19 based on regular blood tests from 5333 patients with various bacterial and viral illnesses, as well as 160 COVID-19-positive patients using the extreme gradient boost machine (XGBoost) and achieved the AUC value of 0.97. According to the significance score of the XGBoost feature, the most beneficial routine blood parameters for the diagnosis of COVID-19 were MCHC, eosinophil count, albumin, INR, and prothrombin activity. 

Rahman et al. [49] used a stacking machine learning model to propose a biomarker-based COVID-19 detection system. This study trained and validated the proposed model using seven different publicly available datasets. White blood cell count, monocyte and lymphocyte percentage, and age parameters were discovered to be important biomarkers for COVID-19 disease prediction. The overall accuracy of the stacking model was 91.45%.

Qu et al. [50] used a logistic regression model to analyze the results of the blood test. The best prognostic indications for severe COVID-19 were lymphocyte count, hemoglobin, and ferritin levels.

The summary of related studies with an emphasis on the significant methods used and the contributions of the studies with their evaluation metrics and values is described in Table 1. The results of the previous study show the applications of single-level and ensemble classifiers. However, some of the shortcomings of existing studies include the challenges of limited dataset samples and imbalance datasets [51], problems with most datasets with aged and male-dominant patient results [52], insufficient clinical data that are useful to improve model classification, challenges of a single data source could lead to model restrictions in generalizability [53], and incomprehensive/inadequate data [54].

Therefore, the need to explore some of the existing feature selection methods for dimensionality reduction is important for an effective classification model [42]. Besides, research focus should be targeted toward analyzing the integrated performance of the new test data using various ML algorithms [35]. Based on some of the existing pitfalls of the previous study, this study presents a unique ensemble method using an automatic feature selection method based on PCA, thus improving the classification of models for efficient COVID-19 detection.

## 3. Proposed Methodology

**Figure 1:**
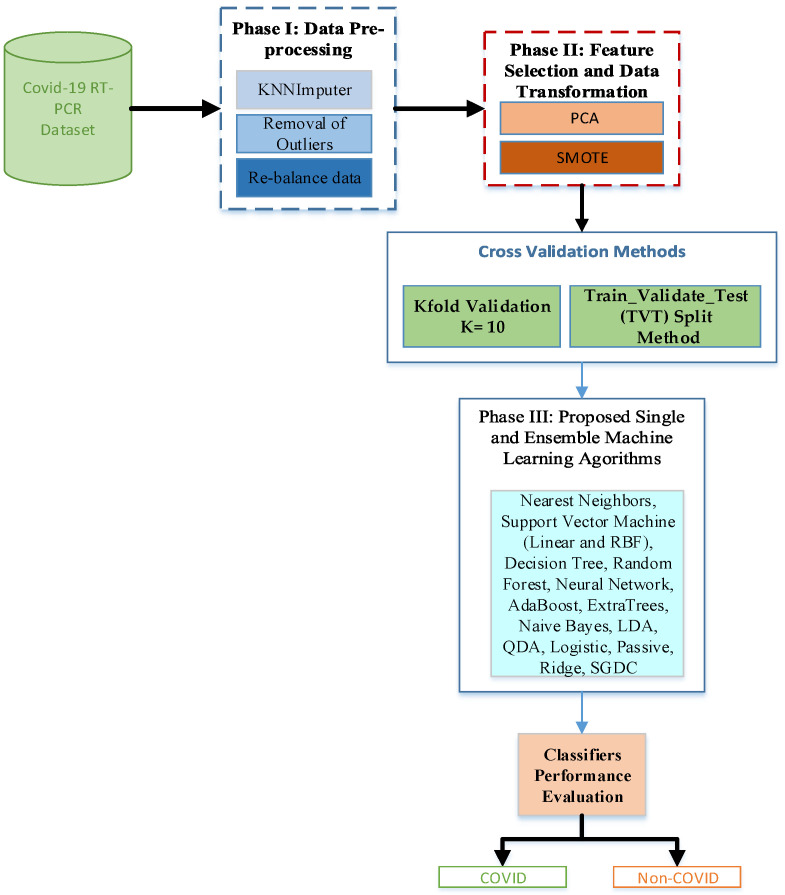
Visual summary of the proposed methodology.

This section discusses in detail the description of the proposed experimental model and the visual summary of the proposed methodology is depicted in Figure 1. Our study applied and investigated the performance of different state-of-the-art ML algorithms, including single and ensemble learning, for effective detection of COVID-19. The proposed system is divided into four categories, and they are fully described in the subsections.

### 3.1. Dataset Description

The dataset used in this study contains 279 cases of patients from San Raffaele Hospital Milan, Italy [27]. It was made accessible by the Italian Scientific Institute for Research, Hospitalization and Healthcare (IRCCS) and annotated with 16 hematochemical values from routine blood tests. The dataset consists of the results of the respiratory tract rRT-PCR test of the samples for 177 positively established cases of COVID-19 and 102 non-COVID-19 cases based on the asopharyngeal swab. The dataset is summarized in Table 2.

### 3.2. Data Preprocessing

This is the first phase of our proposed system, and the concept of data preprocessing has been considered as an important aspect of the generalization performance of supervised ML algorithms [59]. First, we replaced the categorical variable of gender with numerical values (0 for ‘male’ and 1 for ‘female’). We also manually checked all datasets and corrected data typing errors (such as the ‘0–4’ value entered instead of 0.4). After data cleaning, further pre-processing was done to remove outliers. We used the Median Absolute Deviation (MAD)-based outlier removal, which removed the samples that differed by more than three standard deviations from the median value of the variable across the dataset.

In the data preprocessing phase, the need to handle missing values within the dataset is extremely important; thus, we applied the KNN imputation method [60], which allows us to input missing values with the five closest neighbors acting as the best choice, and then input them based on the mean of the non-missing values. We further explore data rebalancing, since the dataset suffers from data imbalance comparing the ratio of positive class to negative class. Previous studies on the impact of class imbalance have shown that if a dataset suffers from imbalance, then classifier biases could lead to classifier biases and hence an increasing misclassification rate and classification model degradation. Based on this, we integrated a synthetic minority oversampling technique (SMOTE) [61], aiming to balance the data by oversampling the minority class.

### 3.3. Feature Selection

**Figure 2:**
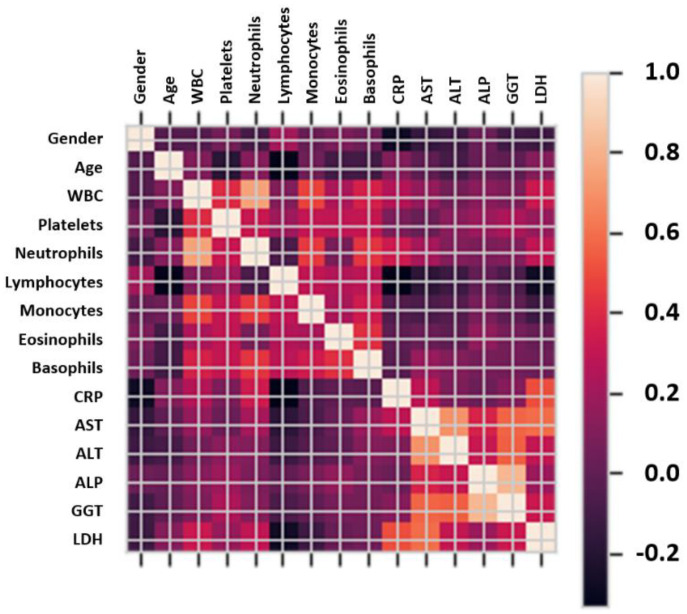
Correlation matrix for the different features of the analyzed blood sample dataset.

The feature selection phase is a crucial stage necessary to select the most appropriate feature representation and improve an ML model. Previous studies have shown that reducing the dimensionality of the data helps reduce data redundancy, avoid noisy data, and improve the performance [62]. This study applied an unsupervised linear transformation technique based on Principal Component Analysis (PCA) to select features with the largest eigenvalues that represent 95% of the variability. The correlation matrix of the different features in the selected datasets is depicted in Figure 2. 

Figure 2 shows in detail the correlation of selected features and the highest feature/parameter is further used and applied to the proposed model. Correlation values between the selected features are used to decide the range of hyperparameters within the learning algorithms.

### 3.4. Cross-Validation Methods

For this study, we applied holdout cross-validation to evaluate the performance of our model as follows. The train-test split function was used from the scikit-learn library to randomly split the data into train/test data samples. The train/test split methods were used to randomly divide the dataset into 80% for training and 20% for testing. We further partitioned the training dataset into the train/validate split using 75% for training and 25% as validation data. Thus, the overall data samples used for training comprise 106 COVID-19 and 62 non-COVID-19 samples, while the validation data comprise 35 COVID-19 and 20 non-COVID-19 samples. To test the performance of our model, the initial holdout of 20% data was used, which consist of 35 COVID-19 and 20 non-COVID-19. The experimental procedure was repeated 10 times, and the performance of each model was measured by calculating the mean average of the recorded scores.

### 3.5. Ensemble Learning

Ensemble learning is a ML approach in which numerous models (dubbed “weak learners”) are trained to tackle the same problem and then combined to achieve superior results [63]. Weak learners (or base models, aka first-stage models) can be used to create more complicated models by merging multiples of them. Most of the time, these base models do not perform well on their own, either because they contain too much bias or too much variation to be robust. The concept behind ensemble techniques is to try to lessen the bias and variance of such weak learners by merging many of them to form a strong learner with superior outcomes. We can generate more accurate or reliable models by combining weak models in the proper way. Base models and a meta-learner (or a second-stage model) that uses base-model predictions are used to design a stacking ensemble model. The base models are trained on the training data and are used to produce predictions. The meta-learner then is trained on the decisions made by base models using previously unseen data to aggregate the base-model predictions. This is done by feeding the meta-learner with the input and output pairs of data from the base learners while aiming to predict the correct output. Therefore, the stacking algorithm has three stages:

1. Construct an ensemble:
  - Select base learners ℬ, which must be different,
  - Select a meta learner ℒ.
2. Train the ensemble:
  - Train each base model on the training dataset D,
  - Cross-validate each base model,
  - Combine the predictions from the base models to form a new training dataset $\hat{D}=\left\{X_{tr},{ℬ}_{1}\left(X_{tr}\right),{ℬ}_{2}\left(X_{tr}\right),\ldots ,{ℬ}_{m}\left(X_{tr}\right)\right\},$ which consists of training inputs $X_{tr}$ and the corresponding predictions by k base models ${ℬ}_{i}\left(X_{tr}\right),\;i=1\ldots k$,
  - Train the meta-learner ℳ on the new dataset $\hat{D}$ to generate more accurate predictions on previously unseen data.
3. Test on new data:
  - Record output decisions from the base models ℬ,
  - Feed base-model decisions into meta-learner ℳ to make final decision.

**Figure 3:**
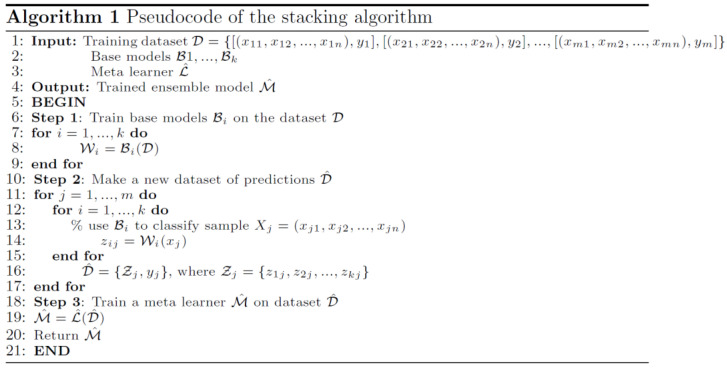
Algorithm of ensemble learning in pseudocode.

The ensemble learning algorithm is summarized in Figure 3. Stacking exploits the capabilities of any best learner. When base classifiers used for stacking have high variability and uncorrelated outputs, the largest improvement in performance is usually made.

### 3.6. Machine Learning Models

We have experimented on 15 ML models, namely KNN, Linear SVM, RBF SVM, Random Forest, Decision Tree, Neural Network (MultiLayer Perceptron), AdaBoost, Extremely randomized trees (ExtraTrees), Naïve Bayes, LDA, QDA, Logistic Regression, Passive Classifier, Ridge Classifier, and Stochastic Gradient Descent Classifier (SGDC). These ML algorithms were used in other classification domains and have achieved the best prediction performances based on their ability to collaborate the benefits of several different algorithms to a more powerful model. To improve generalizability and robustness compared to a single ML algorithm, we applied three different ensemble learners. 

Some of the ML algorithms used in this study are described as follows:

1. The K-Nearest Neighbor (KNN) model has been used effectively in previous studies, especially in solving non-linear problems. It is used to assign the class label according to the smallest distance between the target point and training point(s) in the feature space. The Euclidean distance (ED) is widely used to determine the distance between the target point x and the training point y:
  (1) $$\mathrm{ED}=\sqrt{\sum_{i=1}^{n}{\left(x_{i}-y_{i}\right)}^{2},}$$
2. Support Vector Machine (SVM): This a type of ML technique that has been used effectively in disease detection. This supervised learning algorithm selects the hyper-plane or the decision boundary defined by the solution vector w to determine the maximum margins between training data samples and unknown test data. The most popular variants of SVM are linear SVM and nonlinear SVM with Radial Basis Function (RBF) kernel. The linear SVM binary classifier [64] is expressed in Equation (2). Nonlinear SVM with RBF kernel (Equation (3)) has shown very encouraging outcomes in pattern classification with wide application areas. Considering the training samples ${\left\{y_{i},x_{i}\right\}}_{i=1}^{n},$ with the label $y_{i}\in \left\{-1,+1\right\}$ showing the class of the feature vector $x_{i}\in R^{d}$ in d feature dimensions, the hyperplane H(x) is defined as follows:
  (2) $$H\left(x\right)=w^{T}x+b=\sum_{i=1}^{n}w_{i}x_{i}+b_{i},$$
  (3) $$H\left(x\right)=\sum_{i=1}^{n}w_{i}x_{i}k\left(x,v_{t}\right)+b,$$
3. Naive Bayes (NB) is used for classification where the instances of a dataset are differentiated using specified features. This model is a probabilistic classifier based on strong independence assumptions between features. The mathematical expression for NB classifier is expressed as the best value of P(x/t) and will be predicted value:
  (4) P(x/t)=(P(t⁄x) P(x))/P(t),
  where P(x) and P(t) are the prior probabilities, the posterior probability is represented as P(x⁄t), and P(t⁄x) is the likelihood.
4. Logistic Regression (LR): We presented a logistic regression model to find the optimal regularization strength and thereby prevent overfitting of the model.
5. Random Forest (RF) is an ensemble algorithm that applies the combination of tree predictors with the same distribution for all trees in the forest. Considering the ensemble of classifiers $h_{1}\left(x\right),\;h_{2}\left(x\right),\;\ldots ,h_{k}\left(x\right)$, and with the training set drawn at random from the distribution of the random vector X, Y, the mathematical definition for the margin function is expressed in Equation (5):
  (5) $$mg\left(X,Y\right)=av_{k}I\left(h_{k}\left(X\right)=Y\right)-max_{j\neq Y}av_{k}I\left(h_{k}\left(X\right)=j\right),$$

The generalization error is depicted in Equation (6):

(6) $$PE*=P_{X,Y}\left(mg\left(X,Y\right)<0\right),$$

where I(.) is the indicator function, and $P_{X,Y}$ is the probability over X, Y.

6. Linear Discriminant Analysis (LDA) is a Bayes optimal classifier that is used in many classification problems. LDA finds a one-dimensional subspace in which the classes are separated well. The discriminant function is given by Equation (7):
  (7) $$d_{k}\left(x\right)=2\mu_{k}^{T}\sum_{k}^{-1}X-\mu_{k}^{T}\sum_{k}^{-1}\mu_{k}-2\log\pi \left(k\right),$$

The parameters of these models are summarized in Table 3.

### 3.7. Performance Metrics

The performance of machine algorithms was evaluated using accuracy, false positive rate (FPR), false negative rate (FNR), area under curve (AUC), Matthew’s Correlation Coefficient (MCC), and Cohen’s kappa. The description of the performance metrics used in this study is summarized in Table 4.

### 3.8. Software and Hardware

The ML algorithms were implemented using the scikit-learn 0.19.1, Keras 2.1.6 in Python 3.7 (Python Software Foundation, Wilmington, DE, USA) packages. We have performed all computations in a personal computer with Windows 10 (Microsoft, Redmond, WA, USA), and 64-bit operating system Intel(R) Core(TM) i5-5300U CPU @ 2.30GHz (Intel Corporation, San Francisco, CA, USA), and 8.0 GB RAM.

## 4. Results

This section provides the details of our findings with respect to the performance of each model along with experimental values of our evaluation metrics.

### 4.1. Convolutional Neural Network (First Stage of Ensemble Learning)

This study is built on different neural network models using different levels of hidden layers 1, 2 trained with two different numbers of epochs (10, 50). The activation function used in this study is ReLu and we used Keras and the Tensorflow library. We used a sequential class from the Keras library and further applied an Adam (Adaptive Moment Estimation) optimizer. The train-test split function was used from the scikit-learn library for performing the random splitting of the data into train/test data samples. 

The training of the dataset was done using a simple CNN architecture and this CNN architecture comprises 10 sequential layers, which includes two convolutional 1D layers, followed by 1-D max-pooling and again two convolutional 1D layers followed by 1D max-pooling. The final stage has one flattening layer, and two dense layers, with an activation function of ReLu and Softmax, respectively. Besides, we added a dropout layer between them. The initial input size is the maximum input size for this dataset, which is 12 × 20. We used two different numbers (10 and 50) of epochs to train the network for all the experiments. The training progress for was at its best within Epoch 50. The hyperparameters of the CNN model are presented in Table 5.

### 4.2. Ablation Study of Machine Learning Algorithms (Second Stage of Ensemble Learning)

We implemented the ablation study to determine the best second-stage ML algorithm. Classification is carried out using 15 ML algorithms and, after all training and testing, the results of each experimental performance after 10 runs were analyzed, and the summary of the accuracy performance of each model using the training validation test (TVT) method is presented in Table 6. 

**Figure 4:**
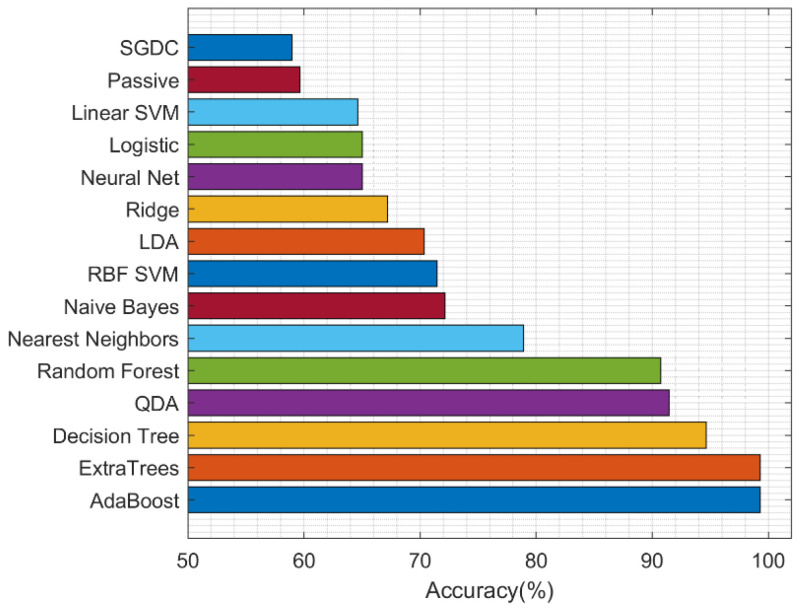
Performance of machine learning models.

The classifier performance is depicted in Figure 4. The experiment was run 10 times and all through the experiment the ensemble classifiers have shown consistency with improved results in the detection of COVID-19. However, the best five performances were achieved by Adaboost, ExtraTrees, Decision Tree, QDA, and random forest models with mean accuracies of 99.28%, and 99.28%, 98.5%, 94.6%, and 92.9%, respectively.

### 4.3. Computational Complexity

The computational complexity of the entire framework is dominated by backpropagation training of the convolutional neural network used in the first stage of the ensemble learning model. The computational cost of the 2D direct convolution is $O\left(F_{I}\times M\times N\times m\times n\times F_{O}\right)$, where M and N are the size of the input feature map, m and n are the size of spatial two-dimensional kernels, and $F_{I}$ and $F_{O}$ are the input and output channels within a layer, respectively [65]. The computational complexity of the best machine learning algorithm used in the second stage of the ensemble learning model (i.e., the ExtraTrees classifier) depend linearly on the number of attributes, which is not high. Formally, it is equal to $O\left(n\times p\times n_{trees}\right),$ where n is the number of training samples, p is the number of features, and $n_{trees}$ is the number of trees.

### 4.4. Statistical Analysis

**Figure 5:**
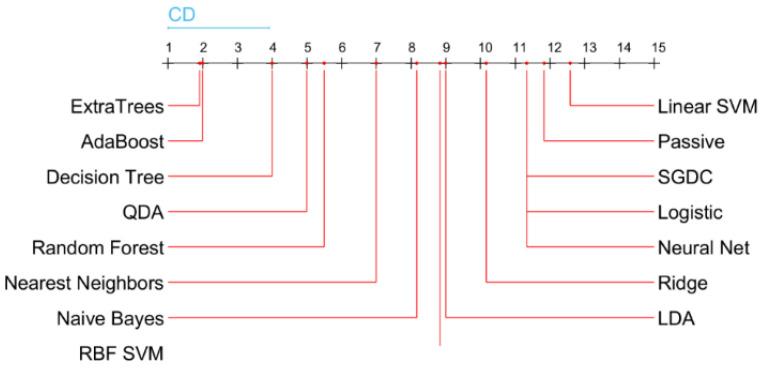
Critical difference diagram of the final-stage classifiers (meta-learners) based on their performances.

To rank the methods, we applied the non-parametric statistical Friedman test and the post hoc Nemenyi test. The Nemenyi test returns the critical difference (CD), which is used to evaluate the significance of the difference between the mean ranks of the methods as presented in Figure 5. If the difference between the mean ranks is smaller than the CD value, then it is considered as not statistically significant. The results of the Nemenyi test show that the ExtraTrees and AdaBoost final-stage classifiers achieved the best performance of 99.28% in accuracy. The result is significantly better than the performance of all other classifiers, except of Decision Tree, which achieved an accuracy of 94.64%.

### 4.5. Comparison with Previous Studies

**Figure 6:**
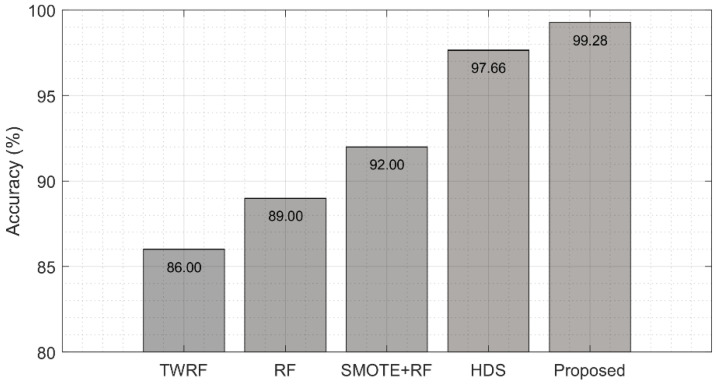
Comparison of results with previous studies. Our proposed model is compared with a three-way random forest classifier (TWFR) approach [27], Random Forest (RF) [67], SMOTE + RF [68], and a Hybrid Fuzzy inference engine and deep neural network (HDS) [66].

For further evaluation of our proposed ensemble learning-based method, we benchmarked the results of our models with previous studies using the same datasets and the same performance metrics. The proposed model shows a significant improvement compared to the existing study using state-of-the-art methods [27,66,67,68] that applied a hybrid fuzzy interference engine and DNN, and a similar study by Brinati et al. [27], which uses a three-way random forest classifier in the prediction of COVID-19 using the RT-PCR dataset. In another study, Chadaga et al. [68] used SMOTE for oversampling, and then evaluated four machine learning algorithms (Random Forest, Logistic Regression, KNN, and Xgboost), while their hyperparameters were optimized using grid search. The best result in terms of accuracy was the 92% achieved with the Random Forest classifier. The summary of related studies with the description of the model type and performance metrics is shown in Figure 6. Our proposed model is compared with a Hybrid Fuzzy inference engine and deep neural network (HDS) approach [66], a three-way random forest classifier (TWFR) approach [27], and Random Forest (RF) [67,68].

## 5. Discussion and Conclusions

The need for early and effective methods for the detection of COVID-19 is extremely important in this era of global pandemic and the application of artificial intelligence methods can significantly improve prediction and assist the physician in the decision-making process. In this paper, the viability and clinical soundness of using blood sample test analysis and machine learning as alternatives to a commonly used RT-PCR test to classify COVID-19-positive patients with were shown. This is particularly useful in countries suffering from scarcity of RT-PCR reagents and specialist laboratories, such as developing ones.

This paper provides simple and interesting stages in the detection of the COVID-19 disease using a small dataset. In addition to the small size of the dataset used in this paper, the problem of missing values, outliers, and class imbalance was also addressed. Our paper explored and analyzed 15 interesting machine learning algorithms, and the experiments were run continuously 10 times on the train-validate-test (TVT) datasets. After 10 runs, we computed the mean metrics and the TVT cross-validation accuracy with the best five models in their descending order: Adaboost, ExtraTrees, Decision Tree, QDA, and random forest with 99.28%, 99.28%, 98.5%, 94.6%, and 92.9%, respectively. In addition, the mean AUC value for ExtraTrees is 99.48%, AdaBoost gave 98.88%, and Decision Tree had 93.72%. 

On the basis of our study, we can argue that our proposed ensemble model outperforms the state-of-the-art methods, as we can see in the next subsection. The COVID-19 early detection ML system based on blood tests offers a quick, simple, and cheaper alternative to imaging scan detection. Our results show the great potential of machine learning with promising results in the detection of the COVID-19 disease. We intend to further explore other medical disease domains using some of our highly performed models in collaboration with deep models in clinical settings.

Some of the limitations and future directions of this study are as follows: only one feature selection technique was applied, thus exploring other feature selection methods can prove useful in improving the results of other machine learning models, thereby increasing classification accuracy. Second, by adopting data augmentation methods, we can aid the performance of training of machine learning methods, with the focus on improving other state-of-the-art single models and, finally, the need to effectively explore more effective deep learning methods to reduce overfitting.

## Figures and Tables

**Table 1:** Summary of related work on COVID-19 identification from blood samples.

Ref.	Methods	Feature Selection Methods	Metrics (Value)	Data Samples (COVID-19 Samples)
[42]	Ensemble learning extra trees, random forest (RF), logistic regression (LR), extreme gradient boosting (ERLX) classifier	Manual	Accuracy: 99.88% AUC: 99.38%, Sensitivity: 98.72% Specificity: 99.99%	5644 (559)
[47]	Categorical gradient boosting (CatBoost), support vector machine (SVM), and LR	Manual	AUC: 89.9–95.8% Specificity: 91.5–98.3% Sensitivity: 55.5–77.8%	5148 (447)
[53]	Ensemble learning with RF, LR, XGBoost, Support Vector Machine (SVM), MLP	Decision Tree Explainer (DTX)	Accuracy (0.88 ± 0.02)	608 (84)
[39]	Artificial Neural Network (ANN) predictive model	Pearson and Kendall correlation coefficient	Area under curve (AUC) values of 0.953 (0.889–0.982).	151
[35]	ANN, RF, gradient boosting trees, LR and SVM	NA	AUC: 0.85; Sensitivity: 0.68; Specificity: 0.85; Brier Score: 0.16	235 (102)
[54]	RF classifier	manual	Accuracy: 96.95%, Sensitivity: 95.12%, Specificity: 96.97%	253 (105)
[55]	ANN, Convolutional Neural Network (CNN), Long-Short Term Memory (LSTM), Recurrent Neural Network (RNN), CNN-LSTM, and CNN-RNN	CNN and LSTM	AUC: 0.90, Accuracy: 0.9230, FI-score: 0.93, Precision: 0.9235, Recall: 0.9368	600 (80)
[56]	SVM, LR, DT, RF and deep neural network (DNN)	Logistic regression (LR)	Accuracy: 91%, Sensitivity: 87%, AUC: 97.1%, Specificity: 95%.	921 (361)
[57]	ANN, CNN, RNN	SMOTE	Accuracy: 94.95%, F1-score: 94.98%, precision: 94.98%, recall: 94.98%, AUC: 100%	600 (80)
[31]	LR	Maximum relevance minimum redundancy (mRMR) algorithm	Sensitivity: 98%, Specificity: 91%	110 (51)
[58]	LR, DT, RF, gradient boosted decision tree	NA	Sensitivity: 75.8%, Specificity: 80.2%, AUC: 85.3%	3346 (1394)

**Table 2:** Summary and description of the dataset.

S/N	Features	Data Types	Number of Missing Values	Mean/Average
1	Gender	Nominal	0	-
2	Age	Numeric	0	61.3
3	WBC ^1^	Numeric	2	8.6
4	Platelets	Numeric	2	226.5
5	CRP ^2^	Numeric	6	90.9
6	AST ^3^	Numeric	2	54.2
7	ALT ^4^	Numeric	13	44.9
8	GGT ^5^	Numeric	143	82.5
9	ALP ^6^	Numeric	148	89.9
10	LDH ^7^	Numeric	85	380.5
11	Neutrophils	Numeric	70	6.2
12	Lymphocytes	Numeric	70	1.2
13	Monocytes	Numeric	70	0.6
14	Eosinophils	Numeric	70	0.05
15	Basophils	Numeric	71	0
16	Swab	Nominal	0	-

^1^ WBC = Leukocytes; ^2^ CRP = C-Reactive Protein; ^3^ AST = Aspartate Transaminases; ^4^ ALT = Alanine Transaminases; ^5^ GGT = γ-Glutamyl Transferasi; ^6^ ALP= Alkaline phosphatase; ^7^ LDH = Lactate dehydrogenase.

**Table 3:** Default parameters values for the machine learning models.

Model	Parameters Values	
KNN	n_neighbors = 3, weights = ‘uniform’, algorithm = ‘auto’, leaf_size = 30, *p* = 2, metric = ‘minkowski’	
SVM	**Linear**	C: 0.025, kernel: [‘linear’]
	**RBF**	C: 1, gamma: 2, kernel: [‘rbf’]
Decision Tree	criterion = ‘gini’, max_depth = 5, max_features = None, max_leaf_nodes = None, min_samples_leaf = 1, min_samples_split = 2, random_state = None, splitter = ‘best’, in_weight_fraction_leaf = 0.0	
Naïve Bayes (Gaussian)	priors = None, var_smoothing = 10^−9^	
Neural Network (MLP Classifier)	activation = ‘relu’, alpha = 1, batch_size = 1024, hidden_layer_sizes = 100, learning_rate_init = 0.001, max_iter = 1000, max_iter = 200, power_t = 0.5, random_state = None, shuffle = True, solver = ‘adam’, tol = 0.0001	
Discriminant Analysis	**Linear**	n_components = None, priors = None, shrinkage = None, solver = ‘svd’
	**Quadratic**	tol = 0.0001, store_covariance = False, reg_param = 0.0, priors = None
Passive	C = 1.0, n_iter_no_change = 5, max_iter = 1000, random_state = None	
Ridge	fit_intercept = True, alpha = 1.0, normalize = False, max_iter = None, random_state = None, solver = ‘auto’,	
SGDC	loss = ‘hinge’, penalty = ‘l2’, alpha = 0.0001, fit_intercept = True, max_iter = 1000,	
Logistic Regression	C = 1.0, cv = None, dual = False, fit_intercept = True, max_iter = 100, penalty = ‘l2’, random_state = None, solver = ‘lbfgs’, tol = 0.0001,	
Ensemble Learner		
Random Forest	max_features = 1, n_estimators = 10, max_depth = 5, criterion = ‘gini’, random_state = None, verbose = 0	
AdaBoost	algorithm = ‘SAMME.R’, learning_rate = 1, n_estimators = 50, random_state = None	
Extra Trees	criterion = ‘gini’, max_depth = None, max_features = 12, min_samples_leaf = 1, min_samples_split = 2, min_weight_fraction_leaf = 0.0, n_estimators = 100	

**Table 4:** Mathematical definition of performance metrics.

Metrics	Definition
Accuracy (Acc)	Acc=((TP+TN)/(TP+TN+FP+FN))
False Negative Rate (FNR)	FNR=(FN/(TP+FN))
False Positive Rate (FPR)	FPR=(FP/(TN+FP))
Matthews Correlation Coefficient (MCC)	$MCC=\left(TP\times TN-FP\times FN\right)/\sqrt{\left(TP+FP\right)\times \left(TP+FN\right)\times \left(TN+FP\right)\times \left(TN+FN\right)}$
Cohen Kappa	$K=\left(P_{o}-P_{e}\right)/\left(1-P_{e}\right)$

TP—true positives, FP—false positives, TN—true negatives, FN—false negatives, $P_{o}$—observed accuracy, $P_{e}$—expected accuracy.

**Table 5:** Description of the convolutional neural network model.

Parameters	Description
Activation Function	Input layer: ReLU
	Hidden layer: ReLU
	Output layer: Softmax
	Loss = sparse_categorical_crossentropy, optimizer = adam,
	Input layer: ReLU
Epochs	10
Epoch 2	50
Batch Size	1024
Dropout ratio (Input)	0.5
Dropout ratio (Output)	0.3

**Table 6:** The results of ablation study: performance of the proposed model using different final stage ML classifiers. Best values are shown in bold.

ML Model	Accuracy (%)	FPR (%)	FNR (%)	AUC (%)	MCC (%)	Kappa (%)
Nearest Neighbors	78.9	39.86	11.02	74.56	51.48	50.8
Linear SVM	64.66	**100**	**0**	50	0	0
RBF SVM	71.44	79.06	1.72	59.62	26.34	21.66
Decision Tree	94.64	9.36	3.2	93.72	88.24	87.94
Random Forest	90.74	22.38	2.2	87.72	79.54	78.64
Neural Net	65.02	99.04	**0**	50.48	3.48	1.18
AdaBoost	**99.28**	2.24	**0**	98.88	98.36	98.32
ExtraTrees	**99.28**	0	1.04	**99.48**	**98.4**	**98.4**
Naive Bayes	72.14	54.14	13.78	66.06	35.48	34.26
LDA	70.34	67.62	8.86	61.8	30.08	26.44
QDA	91.44	18.4	3.3	89.14	81.26	80.46
Logistic	65.02	99.04	**0**	50.48	3.48	1.18
Passive	59.64	60	29.48	55.26	11.48	9.74
Ridge	67.18	92.2	0.52	53.62	17.24	8.82
SGDC	58.96	52.38	34.88	56.36	13.12	15.1

## Data Availability

The dataset used in this study is available from https://zenodo.org/record/3886927#.Yc6feGiOmUk (accessed on 9 February 2022).
